# Structure, Function, and Applications of Soybean Calcium Transporters

**DOI:** 10.3390/ijms232214220

**Published:** 2022-11-17

**Authors:** Bowei Jia, Yuan Li, Xiaoli Sun, Mingzhe Sun

**Affiliations:** Crop Stress Molecular Biology Laboratory, Heilongjiang Bayi Agricultural University, Daqing 163319, China

**Keywords:** calcium, calcium transporter, *Glycine max*, stress response

## Abstract

*Glycine max* is a calcium-loving crop. The external application of calcium fertilizer is beneficial to the increase of soybean yield. Indeed, calcium is a vital nutrient in plant growth and development. As a core metal ion in signaling transduction, calcium content is maintained in dynamic balance under normal circumstances. Now, eight transporters were found to control the uptake and efflux of calcium. Though these calcium transporters have been identified through genome-wide analysis, only a few of them were functionally verified. Therefore, in this study, we summarized the current knowledge of soybean calcium transporters in structural features, expression characteristics, roles in stress response, and prospects. The above results will be helpful in understanding the function of cellular calcium transport and provide a theoretical basis for elevating soybean yield.

## 1. Introduction

Calcium is a kind of the most versatile essential nutrients for plants. Lack of calcium in plant growth tissues results in bloom end rot, tip burn, and bitter pit [1]. Indeed, calcium is a multifunctional divalent cation and acts as a structural component in the cell wall and membranes, an intracellular secondary messenger, a counter-cation for inorganic and organic anions in the vacuole, and an activator of an enzyme [2,3,4]. Mostly, they are absorbed as Ca^2+^ by roots and delivered to shoots via the xylem [1]. In plant cells, the cytosolic Ca^2+^ concentration is maintained at the nanomolar level, but at the millimolar level in the cell wall and vacuole [5,6]. However, the tiny stimulation of various outside environments would trigger intracellular Ca^2+^ increase within seconds/minutes [7]. Thus, the movement of intracellular Ca^2+^ needs fine regulation between extra- or intracellular storage compartments.

Till now, eight different calcium transporters have been gradually identified, including two efflux transporters (Ca^2+^-ATPase, and Ca^2+^/cation antiporter (CaCA)), and six influx transporters (cyclic nucleotide-gated ion channel (CNGC), two-pore cation channel (TPC), glutamate receptor-like protein (GLR), hyperosmolality-gated calcium-permeable channel (OSCA), mid1-complementing activity protein (MCA), and annexins (ANNs)) [8]. Many efforts have been made in the genome-wide identification, structural analysis, transcriptional expression analysis, and functional verification of these calcium transporters in *Arabidopsis thaliana* [9], *Oryza sativa* [10,11], *Zea mays* [12], *Triticum aestivum* [13], *Glycine max* [8], and other species.

Among them, *G. max* is a crucial resource of oil and plant protein. With the fast increase in world population and the great changes in dietary structure, the demand for soybean is increasing yearly [14]. Notably, in the context of COVID-19 and global food price volatility, improving soybean production capacity is an urgent demand in the world. However, the soybean yield is greatly varied due to a diversity of biotic and abiotic stresses, for instance, salt, alkaline, temperature, water stress, insects, and so on [15,16,17]. Thus, in this work, we summarized the current knowledge of soybean calcium transporter in structural features, expression characteristics, and roles in stress response, which will be a benefit to understanding the function of cellular calcium transport and providing a theoretical basis for elevating soybean yield.

## 2. Calcium Efflux Transporters

### 2.1. Characteristic Features and Roles of Ca^2+^-ATPase Involved in Soybean Stress Response

**Figure 1:**
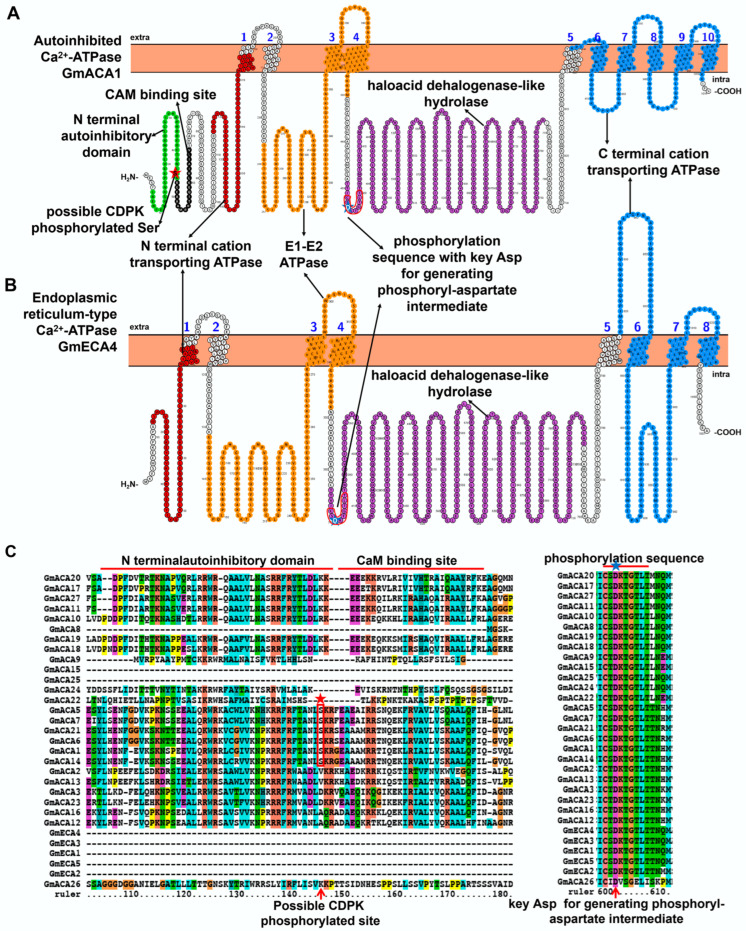
Topology model and homology analysis of soybean Ca^2+^-ATPase, including autoinhibited Ca^2+^-ATPase (ACA) (**A**) and endoplasmic reticulum-type Ca^2+^-ATPase (ECA) (**B**). (**C**) Partial protein sequences alignment of GmACA and GmECA. The red arrows indicate key residues of GmACA and GmECA. The red box in the topology model represents the conserved phosphorylation sequence in the protein sequence alignment. The red star indicates possible CDPK phosphorylated Ser, and the blue star indicates the conserved Asp in the phosphorylation sequence. These protein topology figures were constructed by using Protter [25]. The protein sequences were aligned by using ClustalX1.83 (http://www.clustal.org/download/, accessed on 17 October 2022).

Ca^2+^-ATPase is a member of the P-type ATPase family and can be further classified into two subfamilies (P2A and P2B) [18,19]. In plants, P2A is also called endoplasmic reticulum-type Ca^2+^-ATPase (ECA), and P2B is labeled as autoinhibited Ca^2+^-ATPase (ACA). Both have been found in the plasma membrane, tonoplast, endoplasmic reticulum, and Golgi [20,21,22,23]. Previously, twenty-four *GmACA* and five *GmECA* were identified from the soybean genome [24]. As shown in the homology model and topology structure, both GmACA and GmECA proteins are composed of only one polypeptide, with about 5–10 transmembrane (TM) domains, an N terminal autoinhibitory domain (PF12515), a calmodulin (CAM) binding site, an N terminal cation transporting ATPase (PF00690), an E1-E2 ATPase (PF00122), a haloacid dehalogenase-like hydrolase (PF00702), and a C terminal cation transporting ATPase (PF00689) (Figure 1 and Figure S1). According to their phylogeny, twenty-four *GmACA* were classified into four subfamilies (I-IV), and five *GmECA* were grouped into two subfamilies (I-II). A recent work confirmed this result, but due to the update of the soybean genome database, three more Ca^2+^-ATPase were identified [8]. To facilitate the following description, the name of *GmACA* and *GmECA* discussed here were followed by Sun et al. (2016) (Supplementary Table S1) [24].

Since no corresponding GmACA4 was found in Wm82.a4.v1 (Supplementary Table S1), the remaining GmACA and GmECA protein sequences were downloaded and aligned. As shown in Figure 1, there were no N terminal autoinhibitory domain and calmodulin binding sites in GmECA. The pump activity of the autoinhibitory domain truncation form is higher than full-length ACA, and loses connection to calmodulin [26]. Phosphorylation of the N terminal autoinhibitory domain near the CAM binding site (at Ser45 in GmACA1) by CDPK (Ca^2+^-dependent protein kinase) may inhibit its activity [27] (Figure 1). Therefore, the activity of ACA-subfamily-Group II members (GmACA1/5/6/7/14/21) might be regulated by CDPK. However, the binding of calmodulin will block this phosphorylation. Both GmACA and GmECA contain a conserved phosphorylation sequence CS(/T)DKTGTLT in the haloacid dehalogenase-like hydrolase domain (Figure 1). Among these residues, the highly conserved Asp (D) is a key phosphorylated site during the reaction cycle in all P-type ATPases to generate a phosphoryl-aspartate intermediate (Figure 1) [28].

**Figure 2:**
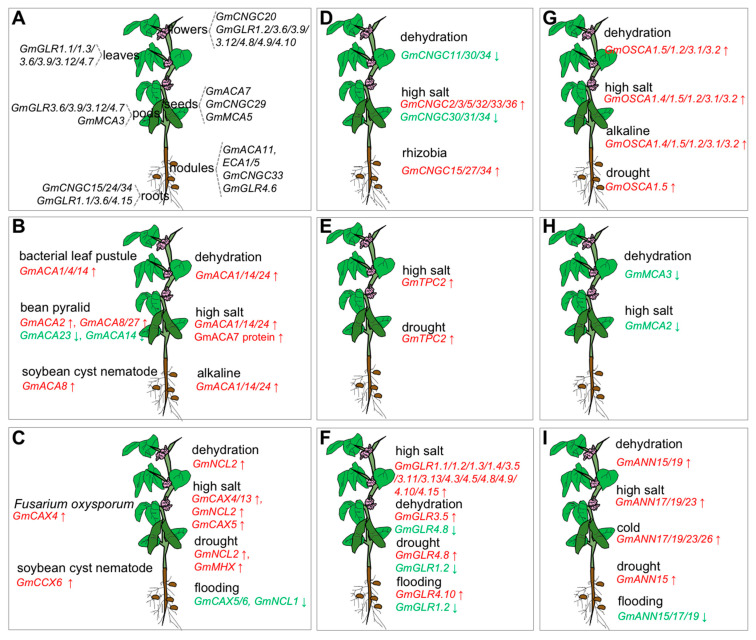
Schematic diagram of expression of eight soybean calcium transporters in soybean tissues (**A**) and under adverse stimuli (**B**–**I**). All these data were collected from published references. The red and upward arrows represent the elevation of expression. The green and downward arrows represent the decrease of expression.

According to the expression data, *GmACAs* and *GmECAs* showed diverse expression patterns (Figure 2A,B). *GmACA1/14/24* was induced by dehydration, high salt, and alkaline stresses [8,24]. GmACA7 protein was especially detected in Wenfeng07 (a salt-tolerant wild soybean) under high salt stress, but not in the salt-sensitive cultivated soybean (Union85140) by using LC-MS/MS [29]. In another work, *GmACA7* was identified as a new QTL associated with the calcium content of soybean seeds [30]. Furthermore, the transcript of *GmACA1/4/14* was high in the BLP (bacterial leaf pustule)-resistant NIL (near-isogenic lines) and induced at 6 h after inoculation in BLP-susceptible NIL [31]. *GmACA2* was highly expressed in resistant line Gantal-2-2 and upregulated in susceptible line Wan82-178 at 48 h after bean pyralid (*Lamprosema indicata*) larvae feeding. *GmACA23* was down-regulated in susceptible line Wan82-178 at 48 h. In the resistant line Gantal-2-2, *GmACA8/27* was highly induced at 48 h after bean pyralid larvae feeding, while *GmACA14* was down-regulated at 48 h [32]. *GmACA8* was up-regulated in both NRS100 (nematode-resistant *soja*, PI578345) and S54 (a soybean cyst nematode race five resistant wild soybean) [33]. *GmACA11* and *GmECA1/5* were identified by LC-MS/MS from soybean symbiosome, suggesting that symbiosome might be a candidate for calcium stores in rhizobia-infected cells [34].

According to the reports, soybean Ca^2+^-ATPases regulates abiotic and biotic stresses at the transcriptional and translational levels. However, only *GmACA1* has been functionally analyzed. In Chung et al. (2000), GmACA1 was proved to be localized at the plasma membrane by using membrane fraction and subcellular localization assay (Table 1). Additionally, there are two Ca^2+^-dependent calmodulin-binding domains (CaMBD) in the N terminus. Yeast mutant complementation experiment verified that GmACA1 functioned as an active Ca^2+^ pump when its N-terminal 85 amino acids were truncated [20]. Later, Sun et al. (2016) found that its wild soybean homologous gene, *GsACA1* functioned as a positive regulator in response to salt and alkaline stresses. *GsACA1* overexpression in alfalfa elevated the activity of Ca^2+^-ATPase and SOD, relieved cell membrane damage, increased the content of proline and chlorophyll, and therefore raised the biomass under salt-alkaline stress [24]. Thus, there is more need to investigate the possible functions of soybean Ca^2+^-ATPases in stress responses and figure out their relationship in the Ca^2+^ signaling pathway.

### 2.2. Characteristic Features and Roles of Ca^2+^/Cation Antiporter Involved in Soybean Stress Response

In stress conditions, the surge of cytosolic Ca^2+^ concentration activates the Ca^2+^ signaling pathway. However, long-term excessive intracellular Ca^2+^ is poisonous to plants. The Ca^2+^/cation antiporter (CaCA) superfamily is widely distributed in living organisms and undertakes the function of Ca^2+^ outward transport and pH regulation [46]. According to the evolutionary analysis, the plant CaCA superfamily is formed of at least four families, including Ca^2+^/H^+^ exchanger (CAX), cation/Ca^2+^ exchanger (CCX), CAX-related Na^+^/Ca^2+^ exchanger like (NCX-like, NCL), and NCX-related Mg^2+^/H^+^ exchanger (MHX) [13,46,47,48,49]. Among them, CAX can form a heterodimer to exert transporting activity and regulate stomata movement and defense responses [50,51]. The others were reported to transport metal ions to participate in stress responses. For example, AtMHX is an H^+^/Mg^2+^ exchanger mediating divalent cations into vacuole [52], AtCCX3 exhibits H^+^-dependent uptake of K^+^/Na^+^ [53], and AtNCL regulates Na^+^ sequestration into vacuole and Ca^2+^ release [54]. Most of them are localized in tonoplast or membrane-contained organelle [55].

**Figure 3:**
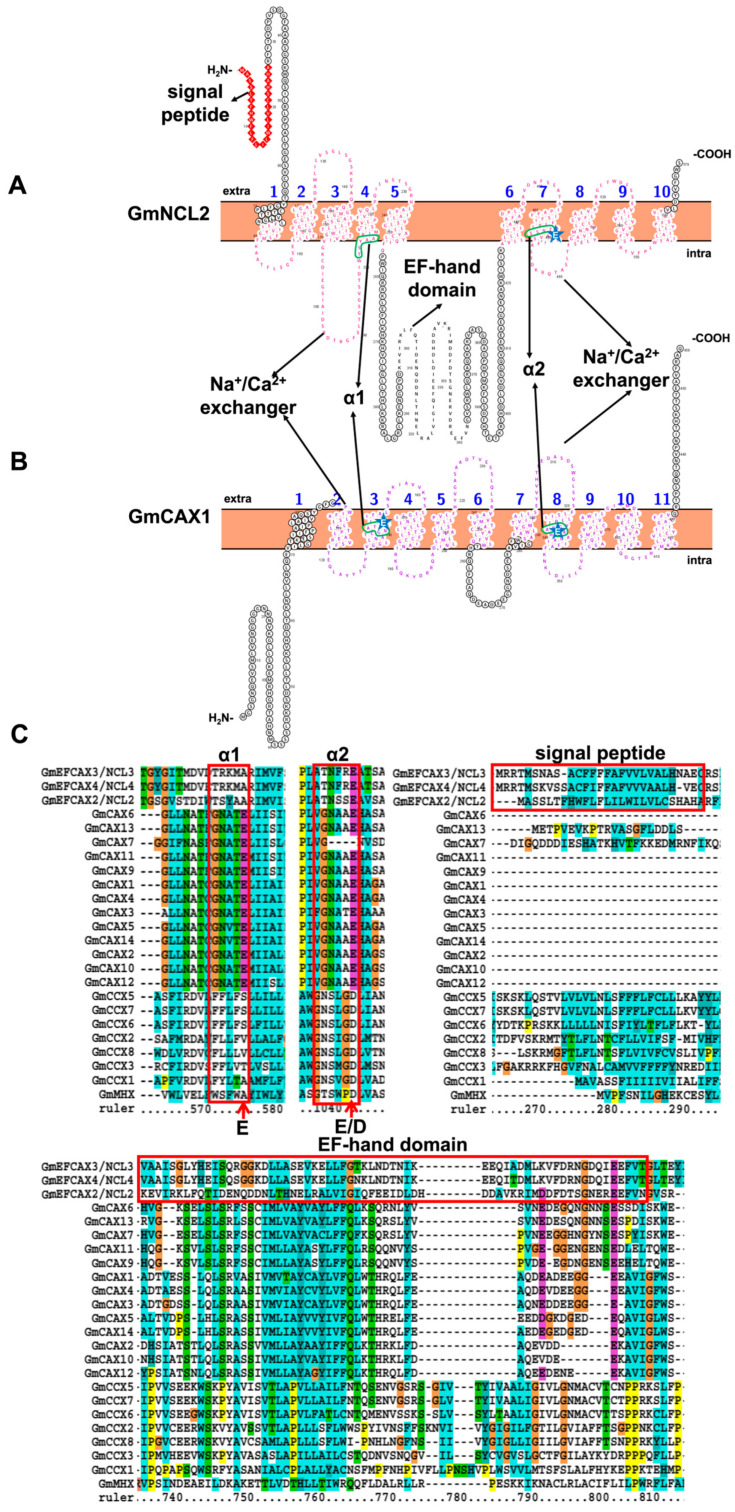
Topology model and homology analysis of soybean Ca^2+^/cation antiporter. (**A**) Topology model of Na^+^/Ca^2+^ exchanger-like (NCL). (**B**) Topology model of Ca^2+^/H^+^ exchanger (CAX). (**C**) Partial protein sequences alignment of soybean Ca^2+^/cation antiporter. The green box in the topology model represents the α-repeat region in the protein sequence alignment. The blue star indicates the conserved Asp or Glu in two α-repeat regions. The red arrows indicate key residues of GmCAX, GmCCX, GmEFCAX\NCL, and GmMHX. These protein topology figures were constructed by using Protter [25]. The protein sequences were aligned by using ClustalX1.83 (http://www.clustal.org/download/, accessed on 17 October 2022).

Based on the recent genome-wide analysis of soybean, a total of twenty-seven CaCA proteins (fourteen CAX, eight CCX, four NCL, and one MHX) have been identified [8,47] (Supplementary Figure S2 and Table S1). Structurally, soybean CaCA proteins share a similar topology with approximately 10-11 transmembrane domains, separated by a large cytosolic loop (an acidic helix), which is essential for Ca^2+^ transport, with a piece of evidence that only the N-terminal half of CAX co-expressed with CAX could activate Ca^2+^ transport (Figure 3) [44]. Indeed, all soybean CaCA proteins have two α-repeat regions that overlap with the Na^+^/Ca^2+^ exchanger domain (PF01699) within TM 2-3 and 7-8 (Figure 3 and Figure S3) [56]. According to the protein sequences alignment, the two α-repeat regions vary a lot among different subfamilies but are conserved within the same subfamily. In fact, GmCAX is conserved with the GNA(/V)TE motif in α1 and the GNAAE motif in α2. GmCCX is conserved with FF(/L/Y)LF(/L/V/T)S(/V/T/A) motif in α1 and NSL(I/M/V)GD motif in α2. Relatively speaking, the last amino acid of the α2 motif is more conservative, either Asp (D) or Glu (E), which is proposed to neutralize the positive charge on Ca^2+^ [47,56]. In the homology models, two α-repeat regions are located near the cell membrane (Supplementary Figure S3). Indeed, there is an additional signal peptide and an EF-hand domain (PF00036) in the NCL family (Figure 3 and  Figure S3). Therefore, they were also called EFCAX.

The expression of soybean *CaCA* have been reported to be regulated by diverse stimuli (Figure 2C). *GmCAX4/13* was induced by salt stress at 6 h, and *GmNCL2* was induced by salt stress and dehydration at 1 h. Only *GmNCL2* and *GmMHX* were upregulated by drought stress. *GmCAX5/6* and *GmNCL1* were enormously decreased at flooding stress. *GmCAX4* was upregulated by *Fusarium oxysporum* infection at 72 h post-inoculation [8]. *GmCCX6* was upregulated in both nematode-resistant soybean NRS100 and S54 [33]. *GmCAX5* (formerly named *GmCAX1*) is the first soybean CaCA gene isolated. RT-PCR assay verified that the expression of *GmCAX5* is ubiquitous in different tissues and induced by PEG (polyethylene glycol), ABA (abscisic acid), and metal ion (Ca^2+^, Na^+^, and Li^+^) treatments. Overexpression of *GmCAX5* in Arabidopsis enhanced high CaCl_2_, NaCl, and LiCl tolerance at the germination stage, with lower Na^+^ and Li^+^ accumulation (Table 1). Unlike tonoplast-localized AtCAX1 and OsCAX1a, GmCAX5 exhibited plasma membrane location, and its Ca^2+^ transport activity still needs further verification [43].

## 3. Calcium Influx Transporters

### 3.1. Characteristic Features and Roles of Cyclic Nucleotide-Gated Ion Channel (CNGC) Involved in Soybean Stress Response

CNGCs are a member of non-selective cation-conducting channels, promoting Ca^2+^ absorption under the regulation of Ca^2+^/CAM and cyclic nucleotide monophosphates (cNMPs). Till now, CNGC proteins have been gradually identified in various green plants, such as 8 in *Physcomitrella patens* [57], 16 in *Oryza sativa* [58], 47 in *Triticum aestivum*, 9 in *Hordeum vulgare* [59], 39 in *Glycine max* [8], 35 in *Nicotiana tabacum* [60], 20 in *Arabidopsis thaliana* [61], 18 in *Solanum lycopersicum* [62,63], and 30 in *Brassica rapa* [64]. According to their phylogeny, all these reported CNGC proteins could be divided into four groups (I–IV) and two subgroups (IVa and IVb). Additionally, they shared highly conserved protein sequence similarity in CNBD. Most CNGCs were found to be located in the plasma membrane (PM), but some were in the endoplasmic reticulum (ER), Golgi, nucleus, and other organelles [65,66]. They were reported to be involved in Na^+^, K^+^, and Ca^2+^ uptake to regulate plant development and stress responses. However, we know little about soybean CNGCs.

**Figure 4:**
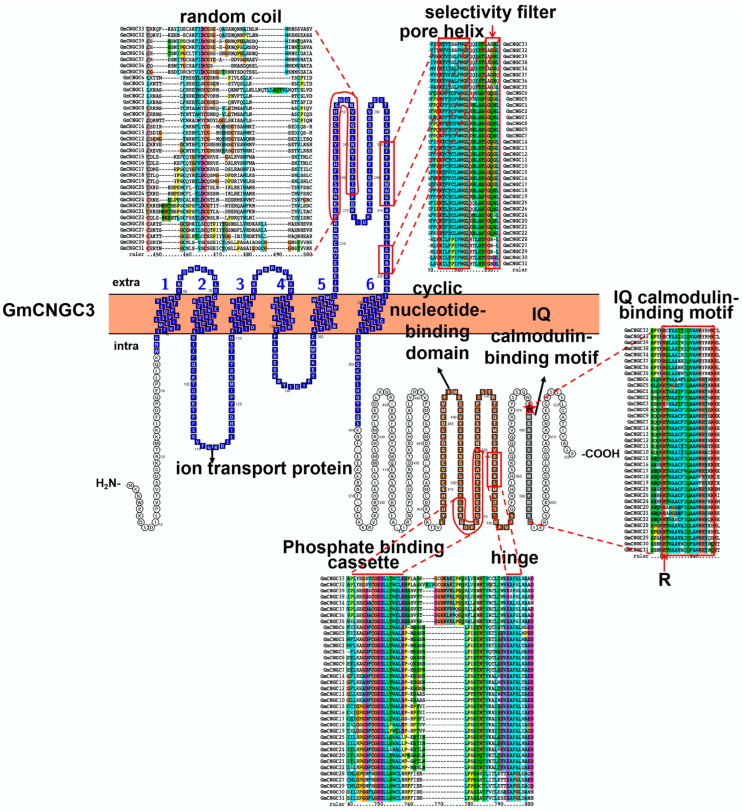
Topology model and homology analysis of soybean cyclic nucleotide-gated ion channel (CNGC). The red star indicates the conserved Arg in IQ calmodulin-binding motif. The protein topology figure was constructed by using Protter [25]. The protein sequences were aligned by using ClustalX1.83 (http://www.clustal.org/download/, accessed on 17 October 2022).

In recent work, 39 soybean CNGCs were identified and divided into group I (9), II (5), III (12), IVa (8), and IVb (5) (Supplementary Table S1) [8]. According to structural analysis, soybean CNGCs possess 6 transmembrane domains in N terminus, ion transport domain (PF00520), C-terminal cyclic nucleotide-binding domain (CNBD) (PF00027), and isoleucine-glutamine (IQ) calmodulin-binding motif (CAMB) (PF00612) (Figure 4). As shown in Figure 4, six transmembrane domains overlap the ion transport domain. After the fifth transmembrane domain, there is a P-loop region which consists of a random coil, a pore helix, and the sixth transmembrane domain (Figure 4). As the homology model depicted, the P-loop region serves as a pathway for cation transportation by forming a tetramer [67] (Supplementary Figure S4). Recently, yeast two-hybrid (Y2H) and bifluorescence complementation (BiFC) assays have verified plant CNGC-CNGC interactions [68,69]. Protein sequence alignment also revealed that soybean CNGCs harbors five selectivity filters in the P-loop region, including GQG, GQN, GQS, G-NL, and AGN triplets amino acids [70]. GQN, GQG, and GQS triplets have been reported to permeate Ca^2+^ [71]. The sequence of CNBD is an essential feature for plant CNGC proteins, with a phosphate-binding cassette (PBC), a hinge region, and a calmodulin-binding domain (CaMBD). The PBC, with conserved GD(/E)ELL motif, is in charge of binding the cNMP ligand and hinge motif via the sugar and phosphate. The hinge region, which contains the AFA(/G/S)L motif, is responsible for ligand selectivity and binding efficiency. For some CNGC proteins, the binding of CaM at the IQ domain could enhance CNGC activity [72]. The conserved Arg in the IQ domain (Arg-X6-Ile-Gln-X-Ala-Trp-Arg) plays a vital role in regulating CNGCs activity (Figure 4) [73].

RNA-seq analysis showed that *GmCNGC2/5/7/8/9/12/13/14/25/26/30/31/37/39* were widely expressed in detected tissues (leaves, flowers, pods, seeds, roots, and nodules). In contrast, *GmCNGC29* showed greater expression during seed development, *GmCNGC20* displayed specific expression in flowers, *GmCNGC33* was only detected in root nodules, and *GmCNGC15/24/34* represented specific expression in roots (Figure 2A). In terms of the stress response, salt stress elevated the expression of *GmCNGC2/3/5/32/33/36*, but repressed the expression of *GmCNGC30/31/34* (Figure 2D). *GmCNGC11/30/34* were also repressed by dehydration. *GmCNGC15/27/34* were upregulated by rhizobia infection. Though the current research about the soybean, CNGC is limited to the transcriptional level; these data provide a theoretical basis for further investigating their performance in soybean development and stress responses. Further, we need to figure out the effect of cNMP and CaM on soybean CNGC and the components and ion specificity of soybean CNGC tetramer.

### 3.2. Characteristic Features and Roles of Two-Pore Cation (TPC) Channel Involved in Soybean Stress Response

**Figure 5:**
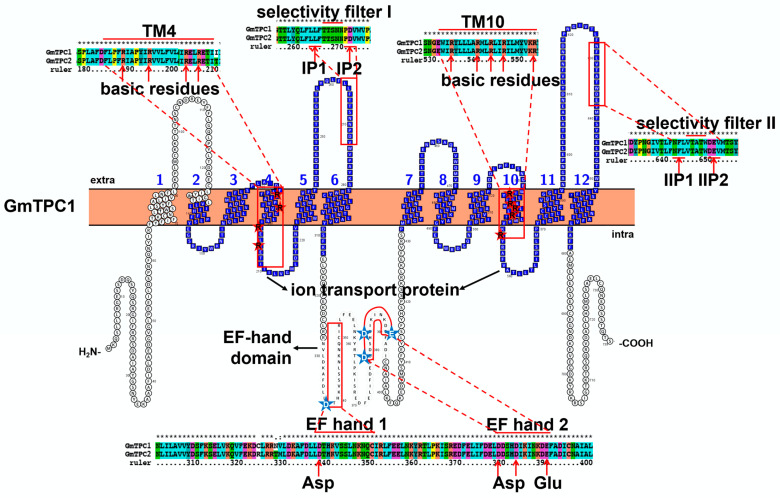
Topology model and homology analysis of soybean two-pore cation (TPC) channel. The red box in the topology model represents the conserved sequence in the protein sequence alignment. The red star indicates conserved basic residue (Arg), and the blue star indicates the conserved Asp or Glu in the EF-hand domain. The protein topology figure was constructed by using Protter [25]. The protein sequences were aligned by using ClustalX1.83 (http://www.clustal.org/download/, accessed on 17 October 2022).

Voltage-gated ion channels contain three related topologies, including single voltage-domain channels, four-domain channels, and TPCs. TPC is a ubiquitously expressed channel protein with very few family members. Plant central vacuole is a huge Ca^2+^ store. Although its Ca^2+^ concentration varies significantly in different tissues, the free vacuolar Ca^2+^ content is controlled in the millimolar range. Many plants have been reported to possess the *TPC* gene, a tonoplast located slow vacuolar (SV) channel, and transports Ca^2+^ from vacuole to cytoplasm. TPC, CNGC, and GLR are the only three ion channels, which function as ligand receptors. Structurally, soybean TPCs contain 12 transmembrane domains, two ion transport domains, and two EF-hand domains (PF13499) (Figure 5 and Figure S5). Similar to CNGC, every six transmembrane domains form an ion transport domain, and a P loop lies between the fifth and sixth transmembrane domains. The first pore loop is similar to Arabidopis TPC channels (with conserved LLFTTSNNPDV motif), while the second is very different (Figure 5). The second filter motif in Arabidopsis is NLLVMGNWQVW, but NFLVTATWDEV in soybean. Since the filter residues have great influences on channel selectivity [74], GmTPC may have different roles from AtTPC in ion transporting. The fourth and tenth transmembrane domains are positively charged, because of the rich basic residues Arg (Figure 5 and Figure S5). However, in the Arabidopsis TPC1 channel, the tenth transmembrane has been proved to be the major voltage-sensing site, and the roles of the fourth transmembrane in voltage sensing were found to be very few [75]. In the homology model, there is a long helix structure, including the sixth transmembrane and partial EF-hand domain in monomer, and GmTPC1 can form a homodimer by crossing two long helix structures (Supplementary Figure S5). Two EF-hand domains are conserved, located on the cytosolic side, and act as the linker of two ion domains. EF-hand 1 consists of DTHKVSSLNKNQC residues and is required for the channel to deal with physiological Ca^2+^ fluctuations. EF-hand 2 (with conserved Asp-X3-Asp-X7-Glu) operates as a Ca^2+^ sensor and regulates the channel open in a voltage-dependent manner when cytosolic Ca^2+^ binds to this site [76] (Figure 5 and Figure S5).

In contrast to mammalian TPCs, plant TPCs localize on the vacuole membrane, and exhibit selective among Ca^2+^, but nonselective among Li^+^, Na^+^, and K^+^ [74]. It has been reported that there was only one TPC in *Arabidopsis thaliana* [77] and *Oryza sativa* [78], but three in *Marchantia polymorpha* [79], two in *Nicotiana tabacum* [76], and two in *Glycine max* [8]. All of them share high protein sequence similarity. Among them, AtTPC1 has been well characterized. AtTPC1 is a tonoplast-located channel response to cytoplasm Ca2+ [80] and is related to the sucrose-induced Ca^2+^ increased, and abscisic acid-induced inhibition of germination. OsTPC1 was identified as a Ca^2+^-permeable channel, which was in charge of Ca^2+^ absorption, and further activates OsMPK2, thus activating ROS-mediated cell death [81]. OsTPC1 was a membrane localization protein in rice cells. However, when heterologously expressed in tobacco cells, OsTPC1 was mainly targeted on the vacuolar membrane [82]. The dual localization of OsTPC1 indicated the diverse membrane protein sorting mechanism among different species and might also create dual functions. Though three MpTPCs are localized on the tonoplast, only MpTPC1 encodes the SV channel according to the vacuole-out recordings assay [79]. Animal TPC activators NAADP (nicotinic acid adenine dinucleotide phosphate), and PI (3, 5) P_2_ (phosphatidylinositol 3,5-bisphosphate) didn’t affect AtTPC1 and MpTPCs. According to RNA-seq data, only *GmTPC2* was ubiquitously expressed across soybean growth and development and induced by salt and drought stress (Figure 2E) [8]. The reported roles of TPC from other species give us a glimpse of the possible function of soybean TPCs, for example, whether animal TPC activators NAADP and PI (3, 5) P_2_ could affect GmTPCs activity, whether GmTPCs is a voltage-activated inward-rectifying Ca^2+^ channel, and whether GmTPCs are tonoplast located protein.

### 3.3. Characteristic Features and Roles of Glutamate Receptor-like (GLR) Protein Involved in Soybean Stress Response

**Figure 6:**
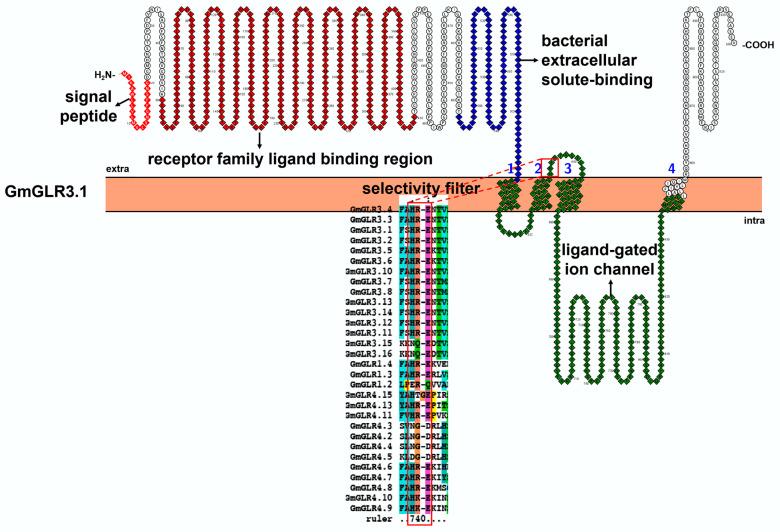
Topology model and homology analysis of soybean glutamate receptor-like (GLR). The red box in the topology model represents the conserved selectivity filter motif in the protein sequence alignment. The protein topology figure was constructed by using Protter [25]. The protein sequences were aligned by using ClustalX1.83 (http://www.clustal.org/download/, accessed on 17 October 2022).

Plant glutamate receptor-like (GLR) genes exist in all photosynthetic organisms and share highly similar amino acid sequences with mammalian ionotropic glutamate receptors (iGluRs) [83]. Further phylogenetic studies suggest that plant GLRs share a common ancestry with animal iGluRs [83,84]. Date to now, a total of 2, 20, 13, 13, 35, 34, and 29 *GLRs* has been identified in the genome of *Physcomitrella patens* [83], *Arabidopsis thaliana* [9], *Solanum lycopersicum* [85], *Oryza sativa* [86], *Glycine max* [8], *Pyrus bretschneideri* [87], *Medicago truncatula* [88], respectively. According to their phylogeny, 35 soybean GLRs could be further phylogenetically divided into four groups (Supplementary Table S1) [8]. It is worth noting that Group IV contains no *A. thaliana* members. In terms of phylogeny, Group IV was far from the other three groups [86]. As reported, soybean GLRs host a long N-terminal extracellular domain (with a signal peptide, a receptor family ligand binding domain (LBD, PF01094), and a bacterial extracellular solute-binding domain (PF00497)), four transmembrane domains, and ligand-gated ion domain (PF00060) (Figure 6). A potential selectivity filter, which is related to the ion selectivity, exists in the ligand gated ion domain, and contains conservative HRE motif (Figure 6 and Figure S6) [83]. According to homology model, GmGLRs exert ion transport functions by assembling four subunits, and four HRE motifs aggregated on the surface of GmGLR tetramer, which may determine its ion transport properties (Supplementary Figure S6).

Both direct biochemical and crystal structural analyses have shown a diversity of amino acid agonists (including Glu, Gly, Ala, Ser, Asn, Cys, and GSH) could increase intracellular Ca^2+^ in whole Arabidopsis seedlings [89]. However, Glu was the most effective agonist for increasing intracellular Ca^2+^ concentration in rice roots and regulating stomatal movement [86,90]. Antagonists of animal iGluRs (including LaCl_3_, GdCl_3_, CNQX, and DNQX) were also active to plant GLRs. Further expression assay suggested that the active agonists and antagonists only affected Ca^2+^ flow, but did not alter the transcript of GLRs [86]. *SlGLR1.1* and *SlGLR3.5* overexpressed Arabidopsis displayed similar morphological phenotype as Ca^2+^ deficiency (with dwarf stature, undeveloped lateral shoots, necrosis of the tips and margins of young leaves) and hypersensitive to additional K^+^/Na^+^. However, Ca^2+^ supplementation rescued this sensitivity. These results may be due to the competitive absorption of K^+^/Na^+^ mediated by *SlGLR1.1* and *SlGLR3.5*, resulting in Ca^2+^ deficiency [85]. In Arabidopsis, *GLR3.5* and *GLR3.7* were involved in Glu-induced stomatal closure. When plants suffered stresses, the content of plant signaling molecule Glu increased and then bound to GLR protein to promote Ca^2+^ influx, and subsequently, CPK (Calcium Dependent Protein Kinase) was activated and phosphorylated SLAC (Slow Anion Channel-Associated), finally leading to stomatal closure. These studies provide a theoretical basis to reveal the function of soybean GLR.

According to RNA-seq data, the expression of *GmGLR* varied a lot under different conditions (Figure 2A,F). *GmGLR1.1/1.3/3.6/3.9/3.12/4.7* were expressed in leaves and pods. *GmGLR4.6* was especially expressed in root nodules. *GmGLR4.15* was specifically expressed in roots. *GmGLR1.3* exhibited predominant expression in leaves. *GmGLR1.2/4.8/4.9/4.10* were mainly found in flowers. These results indicated that *GmGLR4.6* and *GmGLR4.15* might form heteromeric to regulate soybean root architecture, *GmGLR1.2/4.8/4.9/4.10* might share similar functions in flower development. Further transcriptome data reflected that *GmGLR1.1/1.2/1.3/1.4/3.5/3.11/3.13/4.3/4.5/4.8/4.9/4.10/4.15* were upregulated by salt stress, which suggested their similar roles in the salt response. The transcripts of *GmGLR3.5* and *GmGLR4.10* were also separately induced by dehydration and flooding stress. *GmGLR4.8* was elevated by drought stress but decreased by dehydration stress. Both drought and flooding stress the down-regulated expression of *GmGLR1.2*. The above results indicated that *GmGLR1.2* and *GmGLR4.8* might have different response mechanisms in the depicted stress responses. In addition to the above results, little was known about *GmGLRs*. Therefore, further direct physiological, electrophysiological, and biochemical experiments are needed to investigate their function.

### 3.4. Characteristic Features and Roles of Hyperosmolality-Gated Calcium-Permeable Channel (OSCA) Involved in Soybean Stress Response

**Figure 7:**
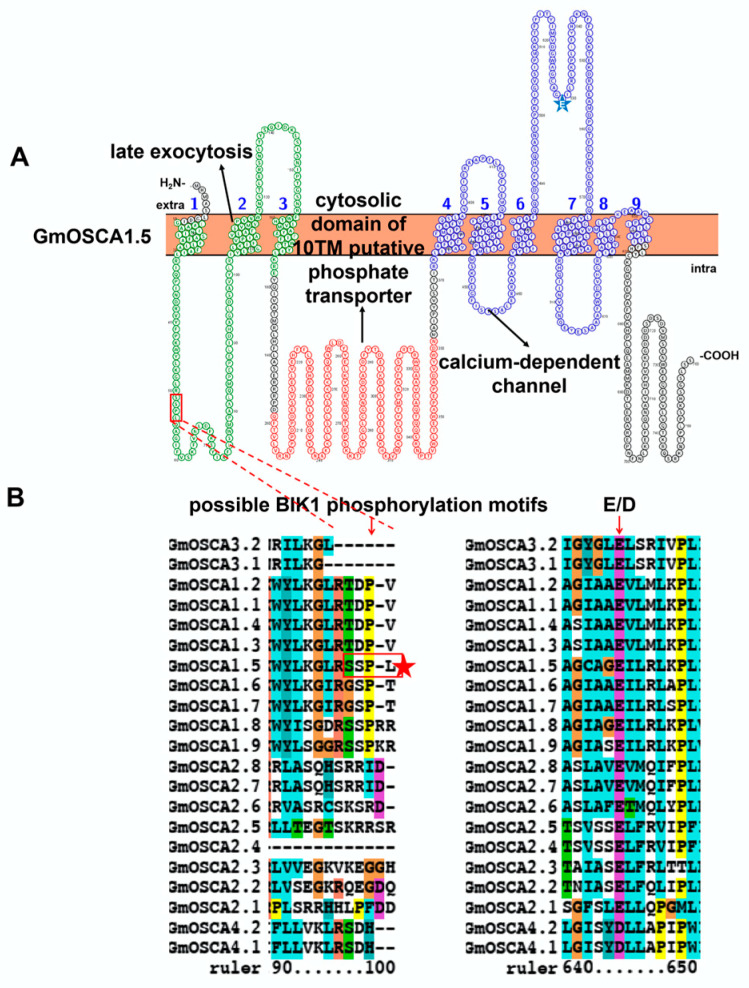
Topology model (**A**) and homology analysis (**B**) of soybean hyperosmolality-gated calcium-permeable channel (OSCA). The red box in the topology model represents the possible BIK1 phosphorylation motif, which is marked with a red box and a red star in the protein sequence alignment. The blue star indicates the conserved Asp or Glu in the calcium-dependent channel domain. The protein topology figure was constructed by using Protter [25]. The protein sequences were aligned by using ClustalX1.83 (http://www.clustal.org/download/, accessed on 17 October 2022).

A mechanosensitive (MS) ion channel is a way for cells to perceive external physical stimulation. Plant MS ion channels consist of five groups: hyperosmolality-gated calcium-permeable channel (OSCA), mid1-complementing activity (MCA), MscS (MS channel of small conductance)-like (MSL), two-pore potassium (TPK), and piezo channel [91]. Among these MS, OSCA (also known as CSC, Calcium permeable Stress-gated cation Channel) is a newly identified osmosensor, which is in charge of hyperosmolality-induced Ca^2+^ increase in Arabidopsis [92,93]. Gradually, 11, 10, and 21 OSCAs were identified in *O. sativa* [10], *Z. mays* [12], and *G. max* [8,94] proteomes, with three conserved domains (namely late exocytosis (PF13967), cytosolic domain 10TM putative phosphate transporter (PF14703), and calcium-dependent channel (PF02714)), and several transmembrane domains (Figure 7). In terms of their phylogenetic analysis, they could be further divided into four main clades. Interestingly, only clade IV was clustered with non-plant species [95]. These results indicated the similar potential functions of OSCA genes from different species within the same clade.

Characterization studies proved that AtOSCA1.2 from clade I, ScYLR241W, and HsCSC1 (*Saccharomyces cerevisiae* and *Homo sapiens* OSCAs) from clade IV displayed conserved osmotically gated Ca^2+^ conductance in Chinese Hamster Ovary (CHO) cells and electrophysiological characteristics in Xenopus oocytes [93]. Asp531 of AtOSCA1.2 was proved to be an essential residue for ion permeation and to participate in cations binding or sequestering (Cryo-EM structure of the mechanically activated ion channel OSCA1.2(E531)). Protein sequence alignment indicated that this site (Asp/Glu) is extremely conserved in Arabidopsis and soybean (Figure 7). Further structure analysis revealed that AtOSCA1.2 forms a homodimer using single-particle cryo-electron microscopy [96]. Consistently, the homology model showed that GmOSCA could form homodimers (Supplementary Figure S7). Another independent work found that plasma membrane protein AtOSCA1.1 (clade I) comprises hyperosmolality-gated calcium-permeable channels, which are in charge of Ca^2+^ increase induced by stimulus [92]. However, they also found that *AtOSCA3.1* (early known as ERD4 (early response to dehydration) from clade III) knockout mutants displayed similar hyperosmolality-induced free calcium increase as wild type, indicating their different role from *AtOSCA1.1* [92]. *AtOSCA1.3* is an immune receptor-associated cytosolic kinase BIK1-activated Ca^2+^-permeable channel, which controls stomatal closure during the immune signalling pathway. This activation relies on the recognition and phosphorylation of the Ser-X2-Leu motif in the N terminus of OSCA1.3 by BIK1 [97]. In soybean, only GmOSCA1.5 has this motif, indicating that it is likely to be the potential substrate of BIK1 (Figure 7). The GhOSCA1.1 virus-induced gene-silenced plants displayed decreased salt and dehydration resistance, with higher water loss, MDA content, and lower SOD activity and proline content, in contrast with control plants [98]. *ZmOSCA2.4* (clade II) overexpressed Arabidopsis exhibited enhanced drought resistance with high chlorophyll and proline content, increased drought tolerance-associated gene expression, and decreased senescence-associated gene expression [99]. These studies provide convenience for uncovering the function of soybean OSCAs.

Two individual studies have reported 21 *GmOSCAs* in the soybean genome (Supplementary Table S1) [8,94]. The expression study of *GmOSCAs* indicated their involvement in alkaline, dehydration, salt, drought, and flooding stresses (Figure 2G). Thirteen of the twenty-one *GmOSCAs* were alkaline stress differentially expressed genes, four were dehydration differentially expressed genes, three were salt stress differentially expressed genes, seven were drought stress differentially expressed genes, and only one was flooding stress differentially expressed genes. In detail, *GmOSCA1.5* was significantly induced by alkaline, dehydration, drought, and salt stresses. *GmOSCA1.2/3.1/3.2* was upregulated by alkaline, dehydration, and salt stresses. *GmOSCA1.4* was induced by alkaline and salt stresses. These expression results provide a theoretical basis for subsequent functional verification. Further specific experimental data is still needed to confirm their function and ion transport characteristics.

### 3.5. Characteristic Features and Roles of Mid1-Complementing Activity (MCA) Protein Antiporter Involved in Soybean Stress Response

**Figure 8:**
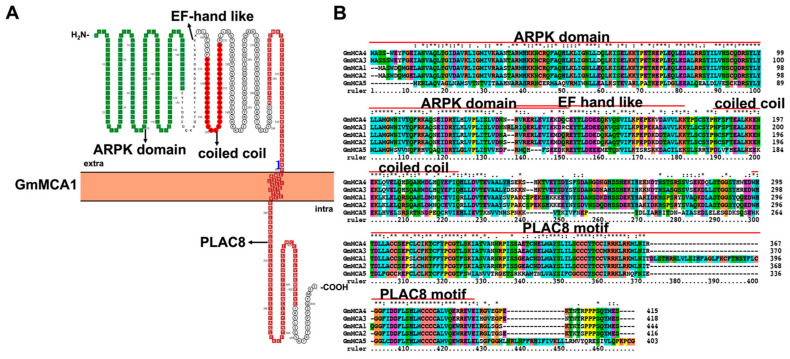
Topology model (**A**) and homology analysis (**B**) of soybean mid1-complementing activity (MCA). The protein topology figure was constructed by using Protter [25]. The protein sequences were aligned by using ClustalX1.83 (http://www.clustal.org/download/, accessed on 17 October 2022).

Mid1-complementing activity (MCA) is also a member of plant MS ion channels [100]. Similar to two Arabidopsis MCA proteins, five GmMCAs were identified with a C-terminus transmembrane, cysteine-rich PLAC8 (PF04749), N-terminus ARPK domain (Amino-terminal domain of Rice putative Protein Kinases, PF19584), and a cytosolic EF hand-like motif (which overlaps with ARPK domain) (Figure 8) [101]. Additionally, a coiled-coil motif is found between the N- and C-terminus (Figure 8) [102]. Homology model analysis suggests that the ARPK domain consists of five helices (Supplementary Figure S8). Further structure truncation analysis illustrated that the N-terminal ARPK domain and EF hand-like motif is necessary and responsible for Ca^2+^ uptake. The C-terminal part is critical for the full activity of AtMCA1, but not for AtMCA2. However, the coiled-coil motif negatively regulates AtMCA1 activity in yeast. The cysteine-rich PLAC8 domain might be responsible for forming tetramer through disulfide bonding or interacting with other proteins [103]. Arabidopsis MCA proteins were confirmed to construct a channel by assembling them into homotetramer. Subcellular location and the yeast mutant *mid1* complementary assay proved that AtMCA1 and AtMCA2 are plasma membrane proteins mediating Ca^2+^ uptake.

The observation of lower Ca^2+^ accumulation in single mutant *mca2* and double mutant *mca1mca2* than in WT and *mca1* indicated the main role of *AtMCA2* in plant root Ca^2+^ uptake. Additionally, the Ca^2+^ absorption is sensitive to ion channel inhibitors GdCl_3_ and LaCl_3_ [104]. Both *AtMCA1* and *AtMCA2* are involved in cold-induced cytoplasm Ca^2+^ increase [105]. Compared to the wild type, mutants *mca1*, *mca2*, and *mca1mca2* displayed dramatically lower cold-induced cytoplasm Ca^2+^ increase. Mutants *mca1mca2* exhibited chilling and freezing sensitivity. In addition, *AtMCA1* and *AtMCA2* overexpression led to hypersensitivity to increased gravity, suppressing the elongation growth at lower gravity levels [106]. Similarly, *NtMCA1* and *NtMCA2* could also rescue the Ca^2+^ uptake activity of yeast mutant *mid1*. Subcellular location and expression data analysis suggested their roles in Ca^2+^-dependent cell proliferation and mechanical stress-induced gene expression by regulating the Ca^2+^ influx [107]. Interestingly, Poaceae has only one *MCA* gene [107]. The same as Arabidopsis and tobacco *MCAs*, *OsMCA1* and *ZmMCA* (also known as *CNR13* and *NOD*) were located on the plasma membrane and could rescue the *mid1* phenotype as well [99,108,109]. Both *OsMCA1* overexpression and suppression lines indicated that *OsMCA1* was a positive regulator in Ca^2+^ uptake and NADPH oxidase-mediated ROS generation induced by hypo-osmotic stress in rice [108,109]. The maize *mca* mutant exhibited deficiency in cell number, size, and differentiation [110].

Five *MCAs* have been identified in soybean with conserved C-terminal PLAC8 domain and only one transmembrane section, which were the same functional domains as reported in AtMCA1/2, NtMCA1/2, OsMCA1, and ZmMCA (Supplementary Table S1). Thus, the functions of reported MCA have shed light on investigating the roles of *GmMCAs*. According to the RNA-seq data, *GmMCA1* and *GmMCA2* were expressed in all detected tissues. The expression of *GmMCA5* was reduced with the development of seeds. The expression of *GmMCA3* was decreased during the growth of pods. *GmMCA3* was down-regulated by dehydration, and *GmMCA2* was down-regulated by high salt stress (Figure 2H) [8]. The above results indicated the potential roles of *GmMCA3* in dehydration response and *GmMCA2* in high salt stress. Indeed, we know nothing about *GmMCAs*. Future, the Ca^2+^ uptake activity of these MCAs and their functions in stress response need further verification.

### 3.6. Characteristic Features and Roles of Annexins Antiporter (ANNs) Involved in Soybean Stress Response

**Figure 9:**
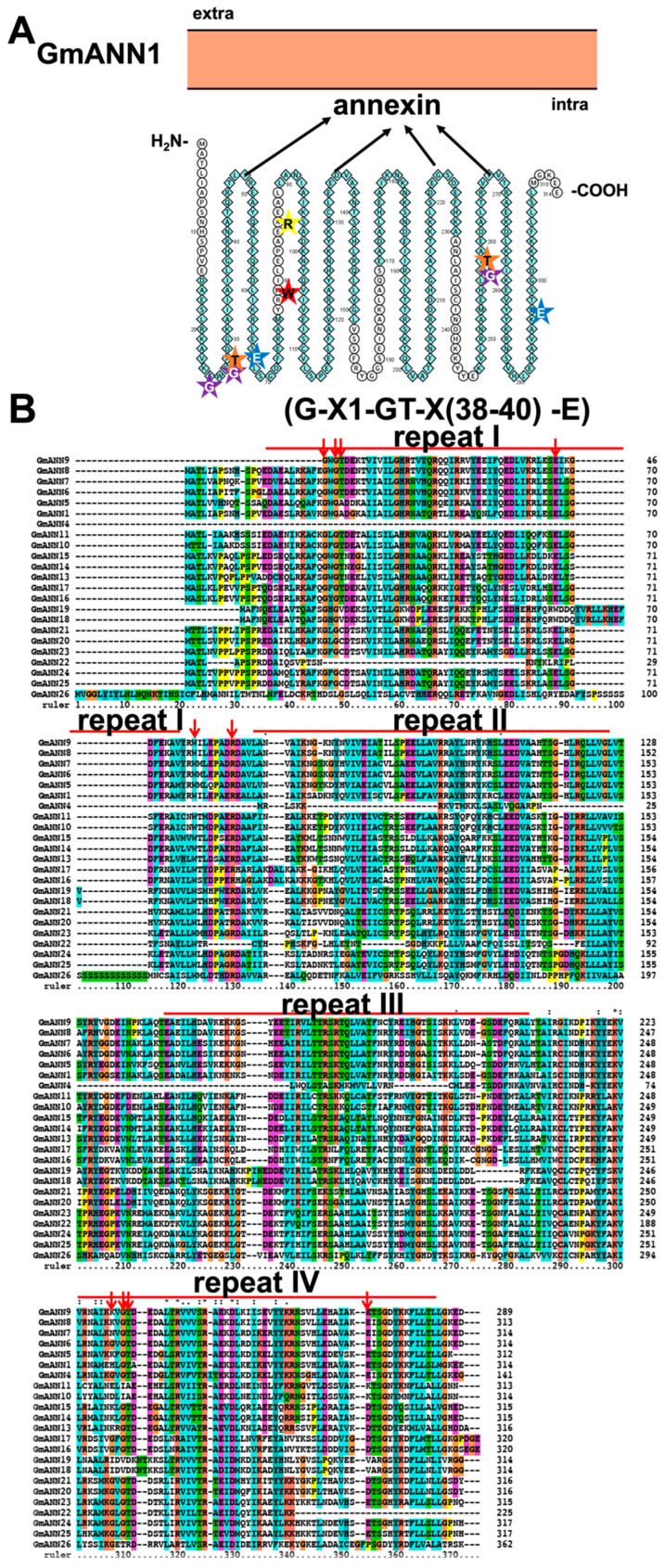
Topology model (**A**) and homology analysis (**B**) of soybean annexins antiporter (ANNs). The red arrows indicate key residues of GmANNs. The conserved residues Ala (G), Thr (T), and Glu (E) in repeats I and IV are indicated by purple, orange, and blue star, respectively. The red star indicates the conserved Trp (W), and the yellow star indicates the conserved Arg (R). The protein topology figure was constructed by using Protter [25]. The protein sequences were aligned by using ClustalX1.83 (http://www.clustal.org/download/, accessed on 17 October 2022).

At first, annexins were identified as novel targets for Ca^2+^ signatures in animal cells [111]. So far, they have been found in most eukaryotes and some prokaryotes, involved in vesicle secretion, ion transport, environmental stimuli, and so on [111,112,113,114]. Plant annexins form a polygene family, which differs from animal annexins in phylogeny and structure [115]. Annexins have been genome-wide identified and analyzed in *A. thaliana* [114], *O. sativa* [11], *G. max* [116,117], and *M. truncatula* [113]. Structurally, they consist of a variable N-terminal and a conserved C-terminal annexin core. Among, the annexin core comprises four similar annexin domains (PF00191, termed as repeat I–IV), which is in charge of Ca^2+^-binding. Each annexin domain consists of a conserved endonexin fold (KG-X-GT-(38-40 residues)-D/E) and five short α-helices (Figure 9 and Figure S9). Till now, a total of 26 soybean annexins have been identified with four annexin repeats (Supplementary Table S1) [8,116]. Sequence analyses revealed that the canonical Ca^2+^-binding sites only exist in repeats I and IV of soybean annexins (Figure 9) [113]. Topological structure and homology modeling analysis suggested that GmANNs are soluble. Four α-helices are arranged in parallel to form a helix-loop-helix bundle structure, and are almost vertically covered by the remaining α-helices (Supplementary Figure S9). GmANNs may be inserted into membranes as oligomers by binding phospholipids in a Ca^2+^-dependent manner [113,118].

Date to now, the roles of annexin have been well characterized in different species. Plant annexins are proposed to take part in the Golgi-mediated formation of the new cell wall, and plasma membrane, with the evidence that plant annexins tend to localize at the periphery of the secretory cells, such as differentiating xylem elements, root cap cells, epidermal cells, as well as the apical meristem cells [119,120]. Functional analyses suggested their multifunction, such as ATPase activity, nucleotide phosphodiesterase activity, F-actin-binding protein, glucan synthesis, peroxidase activity, and channel activity [114,119,121]. The above functions support their vital roles in stress response.

According to Zhu’s work, OsANN1, OsANN3, OsANN4, and OsANN10 are Ca^2+^-binding proteins involved in heat, drought, ABA treatment, and osmotic stresses by modulating ROS balance [119,122,123,124]. Though they were all located on the cell periphery, they exhibited diverse localization in different cells under different conditions. OsANN1-GFP displayed cell periphery in the tobacco leaf epidermal cells and elongation zone of rice root cells, while the GFP signal was found in the cytoplasm in the rice meristematic zone. When subjected to heat stress, OsANN1-GFP was accumulated in the cytoplasm to regulate ROS balance and gene expression. These findings might be a critical process for OsANN1 acting as a positive regulator in heat stress. Indeed, OsANN1-OE lines grew better than WT and RNAi lines under drought stress. OsANN1 also has conformation-dependent ATPase activity. OsANN3 is a positive regulator in response to ABA-dependent drought stress, with increased germination rates, root length, and number, stomatal closure, and reduced water loss in OsANN3-overexpression lines under drought stress [122]. Binding assays confirmed its Ca^2+^-binding activity and the importance of Ca^2+^-binding sites for phospholipid binding activity. The above results provide the possibility for OsANN3 as a Ca^2+^ channel. OsANN4 responds to ABA treatment. OsANN4-RNAi lines showed enhanced ABA sensitivity with lower shoot and root lengths, and accumulated more ROS. Compared to OsANN4-RNAi lines, the presence of ABA promotes Ca^2+^ influx in WT. OsCDPK24 was found to interact with OsANN1 and OsANN4. However, only OsANN4 has been proven phosphorylated by OsCDPK24 at the 13th Ser. It was worth noting that this site did not alter its Ca^2+^-binding ability, but may affect its binding activity by changing OsANN4 conformation. Another annexin, OsANN10, also functions as a Ca^2+^ channel. However, OsANN10 showed different functions from traditional annexins. It plays a negative role in osmotic stress. Lacking *OsANN10* activates the ROS scavenging system, enhances lower MDA content and electrical conductivity, promotes ABA production and stomatal closure, finally maintains more chlorophyll content, and exhibits higher germination rate, plant height, and root length.

In Arabidopsis, salt stress triggered the increase of cytosolic Ca^2+^, which activated the classical SOS pathway, and further inhibited the Ca^2+^ uptake mediated by AtANN4 through negative feedback regulation. Once activated under salt stress, ScaBP8 promotes the interaction between SOS2 and AtANN4 and enhances their phosphorylation, which further enhances its interaction with SCaBP8. Both the interaction and phosphorylation of AtANN4 repress its activity and reduce cytosolic Ca^2+^ concentration [125]. Another work found that AtANN4 could form homodimers and heterodimers with AtANN1 in a Ca^2+^-dependent manner. They cooperatively regulate drought and salt stress responses in a light-dependent way [126]. Under long-day conditions, the loss of AtANN4 or AtANN1 increased Arabidopsis drought and salt stress tolerance, which was strengthened in the *atann1*/*4* double mutant, but AtANN4-OE lines exhibited opposite phenotypes. Cotton annexin GhANN1 plays a positive role in salt stress by increasing ABA accumulation, maintaining the K^+^/Na^+^ homeostasis, and regulating the phenylpropanoid pathway [127]. Transcriptional repressor GhWRKY40-like could bind to the GhANN1 promoter to form a novel GhANN1-ABA-GhWRKY40-like loop to fine-tune cotton salt stress in an ABA-dependent pathway.

Though the study of soybean annexin was still limited to gene identification, expression analysis, and functional verification, the structural similarity sheds light on inferring the operations of GmANNs from the reported functions of AtANN, OsANN, and GhANN. The expression of *GmANN17/19/23/26* displayed diverse organ-specific expression patterns, and they were upregulated by drought and ABA (Figure 2I). *GmANN17/19/23/26* were induced by cold, and *GmANN17/19/23* were involved in high salt stress [116]. In another work, *GmANN19* was induced by salt and dehydration stresses. *GmANN15* was upregulated by salt, dehydration, and drought stresses, while *GmANN15/17/19* were all down-regulated by flooding stress [8]. The above results indicated that *GmANN17/19* might be the critical gene in response to multiple stresses. Additionally, these GmANNs might be assembled into homodimers or heterodimers to exert functions in the same pathway, similar to AtANNs. They also might be similar to OsANNs and exhibit functional differentiation. In drought-sensitive genotype Valder, GmANN15 protein was increased under mild drought stress but decreased under severe drought conditions (Table 1). However, no significant differences were found in drought-tolerant genotype G2120 under these conditions [45]. Thus, these studies suggested a vital role of *GmANN15* in stress response, especially providing further evidence for diverse drought response mechanisms in tolerant and sensitive genotypes. To further uncover the role of GmANNs in plant stress response, whether GmANNs function as channels or by forming homo/hetero-dimers needs to be confirmed. Their interacting protein kinases, transcription factors, and other proteins need to be excavated as well.

## 4. Conclusions and Prospect

Soybean is one of the most noteworthy beans around the world, which is the primary resource of our daily soybean products, edible oil, industrial and medical oil, and high-quality protein feed used by animal husbandry. However, soybean yield is limited by environmental stimuli, such as temperature, water, saline, alkaline, fertilizer, diseases, pests, and so on. As calcium-loving crops, calcium plays significant roles in increasing soybean output and response to adverse stresses [128,129]. In addition to acting as an essential macronutrient for plant growth and development, calcium is the core ion in the complex signaling pathways. The wave of cytosolic calcium concentration reflects the changes in the environment.

**Figure 10:**
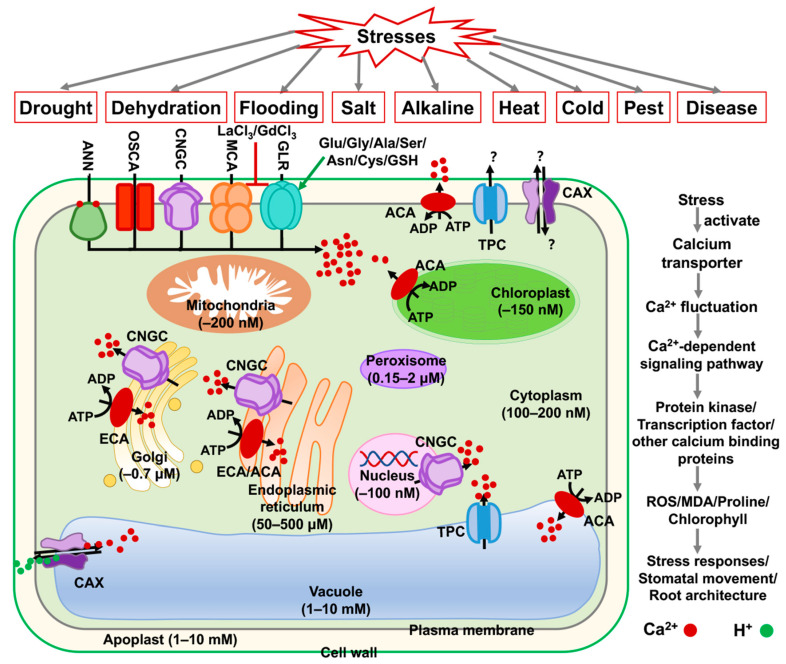
A summarized working model reflecting diverse soybean Ca^2+^ transporters and Ca^2+^ concentrations in different cellular compartments of a soybean cell. The various Ca^2+^ concentration in different organelles is mainly regulated by two efflux transporters (Ca^2+^-ATPase (ACA and ECA), and Ca^2+^/H^+^ antiporter (CaCA)), and six influx transporters (cyclic nucleotide-gated ion channel (CNGC), two-pore cation channel (TPC), glutamate receptor-like protein (GLR), hyperosmolality-gated calcium-permeable channel (OSCA), mid1-complementing activity protein (MCA), and annexins (ANNs)). The red dot is represented Ca^2+^, and the green dot is represented H^+^. GdCl_3_ and LaCl_3_ are inhibitors of GLR and MCA. Glu, Gly, Ala, Ser, Asn, Cys, and GSH are amino acid agonists of GLR.

Thanks to the release of soybean genome sequence data, two calcium efflux transporters (Ca^2+^-ATPase, and CaCA) and six calcium influx transporters (CNGC, TPC, GLR, OSCA, MCA, and ANNs) have been identified (Figure 10). They are all membrane-localized proteins and responsible for the absorption and excretion of Ca^2+^ between various organelles. Indeed, CNGC, TPC, GLR, and ANN were reported to be involved in mediating other metal ions (such as Li^+^, Na^+^, and K^+^) as well. Therefore, further direct evidence is needed to verify their ion transport features. Technical advances enabled us to monitor intracellular calcium fluctuation in real-time, such as non-invasive micro-test technology (NMT), and patch clamp technique. What’s more, two photon-total internal refraction fluorescence (TIRF) microscopy and stimulated emission depletion (STED) microscopy can visualize Ca^2+^ changes within a single cell with the help of Ca^2+^ indicators (such as Calcein). Also, we can generate direct mutation of key functional residues of these calcium transporters involved in the binding or transport selectivity by using the CRISPR/Cas system, which offers a quick and better way to investigate the calcium transport in soybean rather than testing their transport function in a heterologous system [130,131]. Structurally, CAX, CNGC, GLR, MCA, and OSCA from rice and Arabidopsis were reported to exert their function by forming polymers. These findings provide a basis for revealing the mechanism of soybean calcium transporters. Thus, further native-PAGE, size exclusion chromatography (SEC-HPLC), yeast-two hybrid (Y2H), bimolecular fluorescence complementation (BiFC), co-Immunoprecipitation (CoIP), and luciferase complementation assay (LCA) can be applied to verify this.

Though the transcripts of some calcium transporters were found to be regulated by biotic and abiotic stresses, only GmCAX5 and GmACA1 have been functionally analyzed. Future, there is still a lot we need to investigate and verify. For example, whether these calcium transporters function by forming homo- or heterodimers, their relationship with transcription and protein phosphorylation in soybean, their roles in increasing soybean yields, and so on. Also, more calcium transporters are likely to be identified through genome studies. Currently, the research on soybean calcium transporters provides us with valuable genetic resources and new ideas to improve soybean output. Furthermore, we can apply CRISPR-Cas technology to design soybeans with high yield, high calcium content, and strong stress resistance, which will be of great practical significance to promote the increase of soybean yield [130,131].

## Figures and Tables

**Table 1:** List of soybean calcium transporters involved in reported stress response and physiological functions.

Classification	Gene Name	Gene ID Wm82.a4.v1	Subcellular Localization	Arabidopsis Orthologs	Arabidopsis Orthologs Subcellular Localization	Observations
Ca^2+^-ATPase	*GmACA1*	Glyma.01g193600	Plasma membrane [20]	AT4G37640 ACA2	Endoplasmic reticulum [27,35]	dehydration, high salt, alkaline, bacterial leaf pustule [8,24,31]
	*GmACA2*	Glyma.02g186100	Not reported	AT3G57330 ACA11	Vacuole [36]	bean pyralid [32]
	*GmACA4*	No correspondence	Not reported	AT3G63380 ACA12	Plasma membrane [37,38]	bacterial leaf pustule [31]
	*GmACA7*	Glyma.06g046000	Not reported	AT1G27770 ACA1	Chloroplast [39]	high salt, bean pyralid, and a new QTL associated with the calcium content of soybean seeds [29,30]
	*GmACA8*	Glyma.07g004300	Not reported	AT3G21180 ACA9	Plasma membrane [40]	bean pyralid, nematode [32,33]
	*GmACA11*	Glyma.09g061200	Not reported	AT5G57110 ACA8	Plasma membrane [37,41]	soybean symbiosome [34]
	*GmACA14*	Glyma.11g048300	Not reported	AT4G37640 ACA2	Endoplasmic reticulum [27,35]	dehydration, high salt, alkaline, bacterial leaf pustule, and bean pyralid [8,24,31,32]
	*GmACA23*	Glyma.19g136400	Not reported	AT2G41560 ACA4	Vacuole [22]	bean pyralid [32]
	*GmACA24*	Glyma.19g159900	Not reported	AT3G63380 ACA12	Plasma membrane [37,38]	dehydration, high salt, and alkaline [8,24]
	*GmACA27*	Glyma.15g167500	Not reported	AT4G29900 ACA10	Not reported	bean pyralid [32]
	*GmECA1*	Glyma.03g175200	Not reported	AT1G07670 ECA4	Plasma membrane, TGN, Cytosol [42]	soybean symbiosome [34]
	*GmECA5*	Glyma.19g175900	Not reported	AT1G07670 ECA4	Plasma membrane, TGN, Cytosol [42]	soybean symbiosome [34]
Ca^2+^/cation antiporter	*GmCAX5*	Glyma.07g149600	Plasma membrane [43]	AT3G51860 AtCAX3	Tonoplast [44]	PEG, ABA, Ca^2+^, Na^+^ and Li^+^ treatments [43]
Annexins	*GmANN15*	Glyma.08g136200	Not reported	AT5G65020 annexin 2	Not reported	salt, dehydration, drought, and flooding stresses [8,45]

## Supplementary Materials

The following supporting information can be downloaded at: https://www.mdpi.com/article/10.3390/ijms232214220/s1.
