# Supplementary material for: Structure, Function, and Applications of Soybean Calcium Transporters
Source: Int J Mol Sci. 2022 Nov 17;23(22):14220. doi: 10.3390/ijms232214220 (PMC9693241; doi:10.3390/ijms232214220)
Supplement: Supplementary file 1 [file ijms-23-14220-s001.zip › Supplementary Figure S1-S9-20221113.pdf]

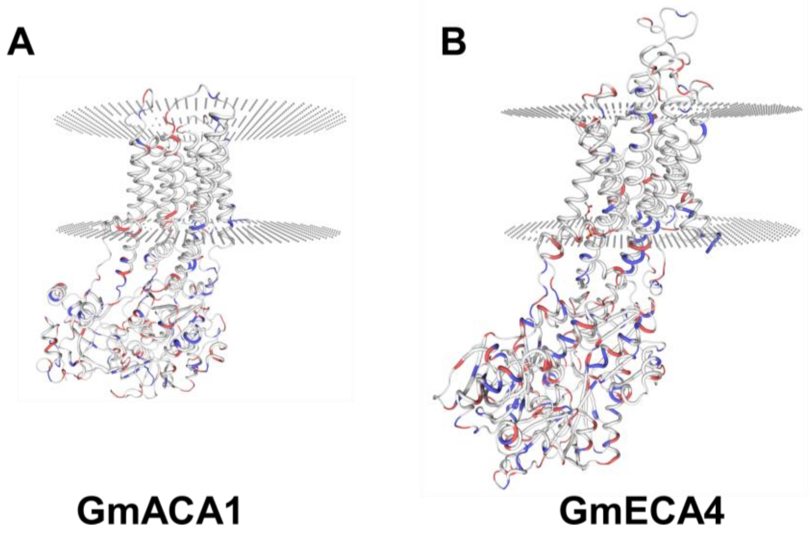

Supplementary Figure S1. Homology model of soybean Ca^{2+}-ATPase, including GmACA1 (A) and GmECA4 (B). They were built by using SWISS-MODEL. The red represents the acid residues, and the blue marks the basic residues. The gray dot plane indicates the cell membrane.

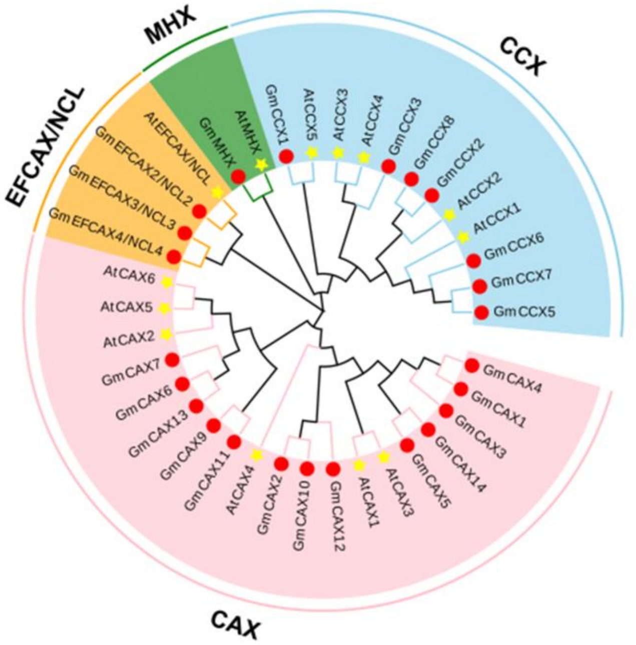

Supplementary Figure S2. The evolutionary relationship of soybean and Arabidopsis CaCA members. This Neighbor-Joining tree was constructed by using MEGA X software method with 1000 bootstrap replications. 24 CaCA members could be further divided into four subfamilies, including Ca^{2+}/H^+ exchanger (CAX), cation/Ca^{2+} exchanger (CCX), CAX-related Na^+/Ca^{2+} exchanger like (NCX-like, NCL), and NCX-related Mg^{2+}/H^+ exchanger (MHX). The red circle represents soybean members and the yellow five-pointed star indicates Arabidopsis members.

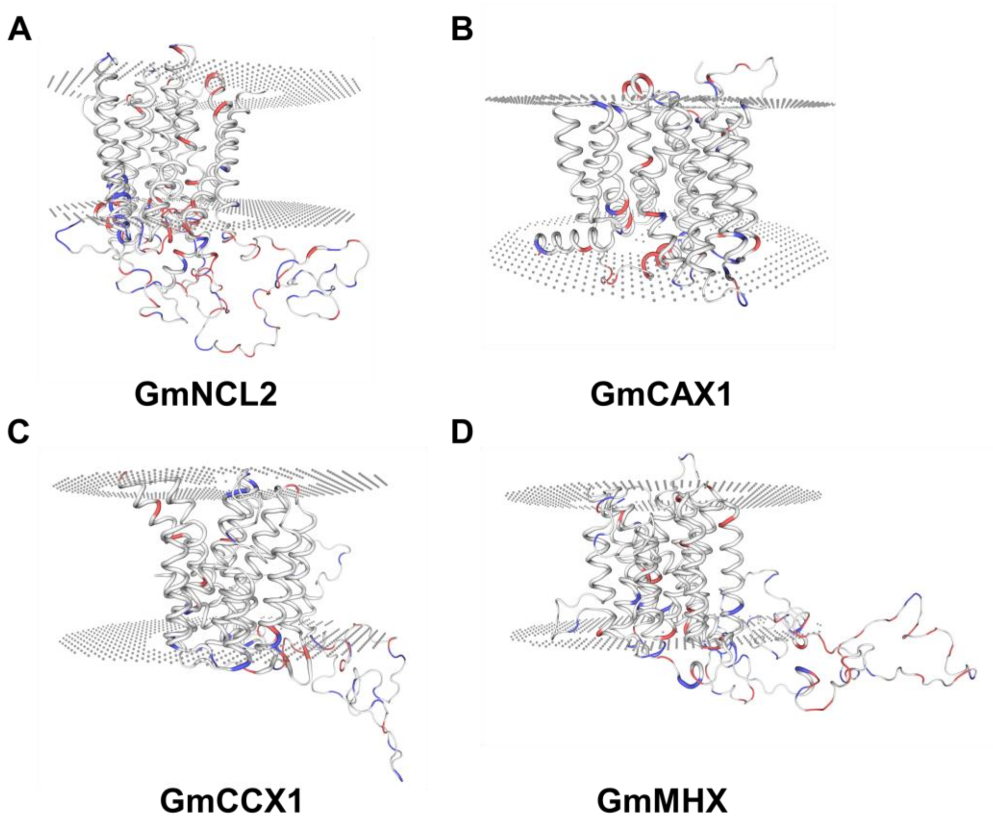

Supplementary Figure S3. Homology model of soybean CaCA, including GmEFCAX\NCL2 (A), GmCAX1 (B), GmCCX1 (C) and GmMHX (D). They were built by using SWISS-MODEL. The red represents the acid residues, and the blue marks the basic residues. The gray dot plane indicates the cell membrane.

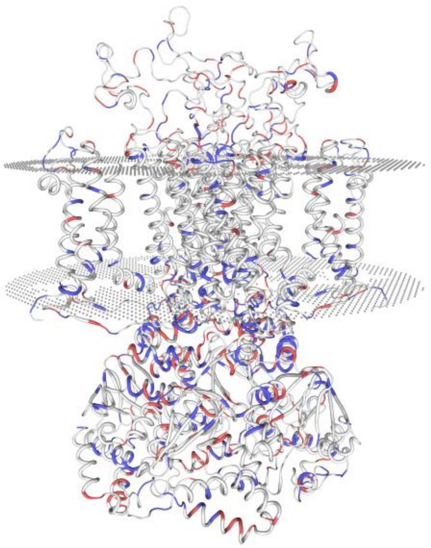

Supplementary Figure S4. Homology model of GmCNGC3 homo-tetramer. It was built by using SWISS-MODEL. The red represents the acid residues, and the blue marks the basic residues. The gray dot plane indicates the cell membrane.

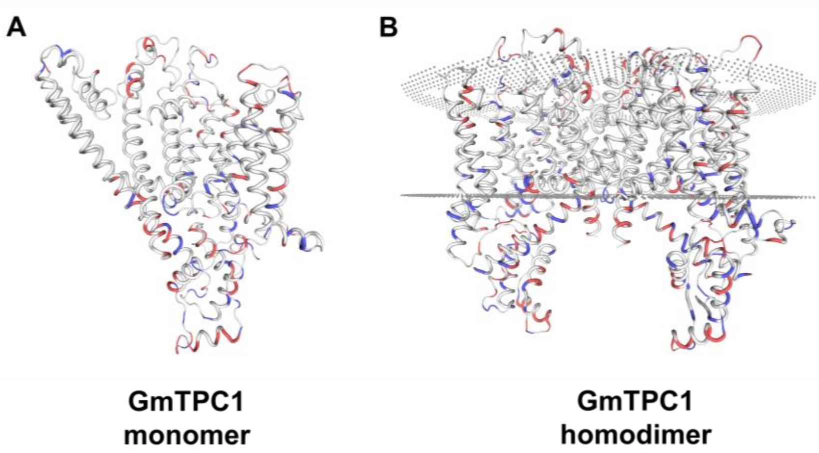

Supplementary Figure S5. Homology model of GmTPC1, including monomer (A) and homodimer (B). They were built by using SWISS-MODEL. The red represents the acid residues, and the blue marks the basic residues. The gray dot plane indicates the cell membrane.

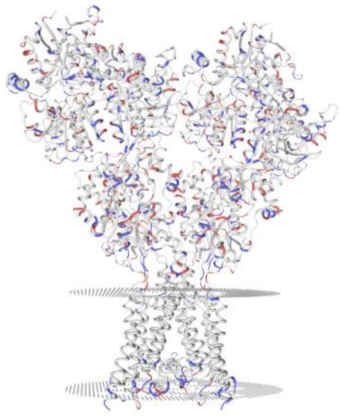

Supplementary Figure S6. Homology model of GmGLR3.1 homo-tetramer. It was built by using SWISS-MODEL. The red represents the acid residues, and the blue marks the basic residues. The gray dot plane indicates the cell membrane.

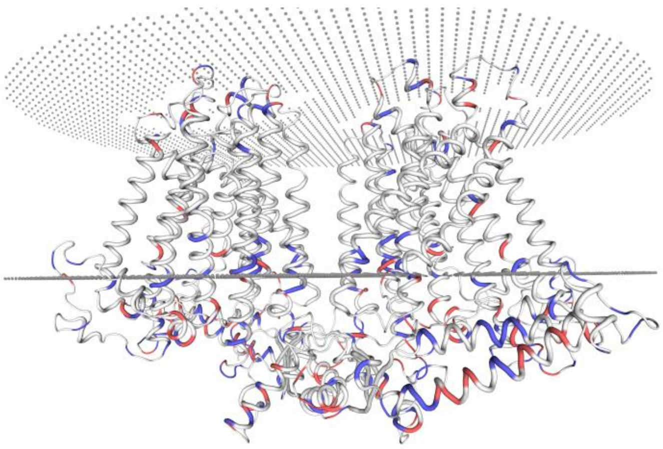

Supplementary Figure S7. Homology model of GmOSCA1.5 homo-dimer. It was built by using SWISS-MODEL. The red represents the acid residues, and the blue marks the basic residues. The gray dot plane indicates the cell membrane.

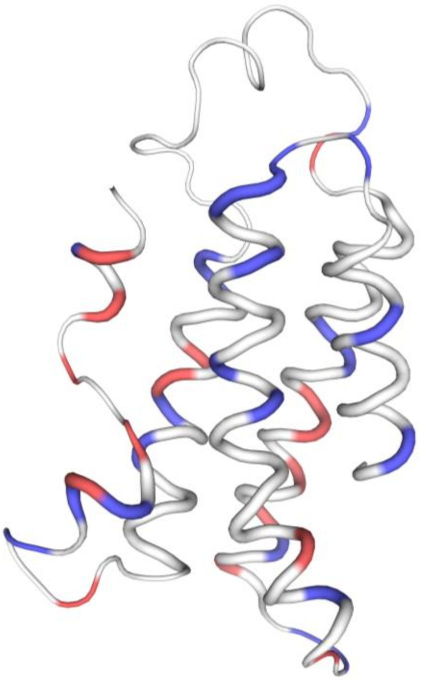

Supplementary Figure S8. Homology model of the ARPK domain in GmMCA1. It was built by using SWISS-MODEL. The red represents the acid residues, and the blue marks the basic residues.

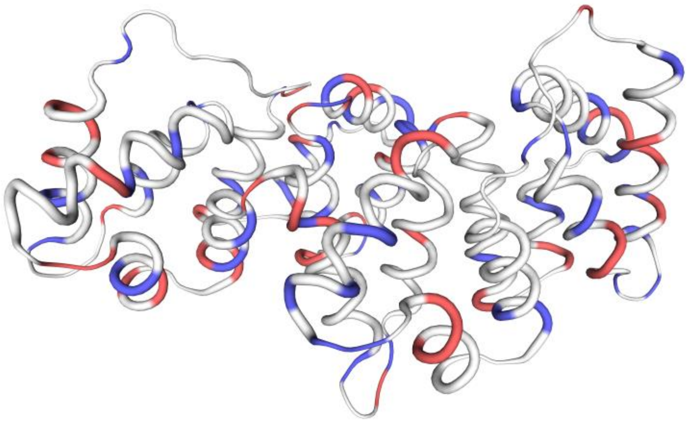

Supplementary Figure S9. Homology model of GmANN1. It was built by using SWISS-MODEL. The red represents the acid residues, and the blue marks the basic residues.
